# Nanoscale in Photocatalysis

**DOI:** 10.3390/nano7040086

**Published:** 2017-04-17

**Authors:** Zhaohui Wang, Hongqi Sun

**Affiliations:** 1 International Centre for Balanced Land Use (ICBLU), The University of Newcastle, Callaghan, NSW 2308, Australia; 2 School of Engineering, Edith Cowan University, 270 Joondalup Drive, Joondalup, Perth, WA 6027, Australia

Rationally harvesting sunlight to carry out chemical reactions, for example, photochemistry and photocatalysis, has appeared as a beautiful episode within the long history of solar-energy utilization by human beings. Before solar-driven chemical processes approach the scale of solar panel or solar thermal application, considerate efforts are still required from effective collaborations among academia, industries, and governments. Over past few decades, the photocatalysis frontier has been continuously explored by means of interdisciplinary research. Emerging understanding in the nanoscale is most likely to lead photocatalysis into the future.

In recognition of these challenging issues, this special issue is a call for studies on the salient features of photocatalysts at nanoscale and the associated mechanisms to bring forth the next generation of photocatalysis, with implications for remediation, solar fuels and sustainability as a whole. The goal of this special issue is to present “dedicated collections” showcasing advances emerging in the research frontier involving any aspect of the *Nanoscale in Photocatalysis*. We are committed to providing sophisticated yet balanced contributions focusing on the state-of-the-art in photocatalysis findings from leading research groups. 

Combining various light-absorbing materials for the development of semiconductor heterojunctions or hybrid photocatalysts is a very effective strategy to extend the light absorption threshold and to optimize charge carrier separation. This special issue, with a collection of 10 original contributions of research articles or review papers, presents the most recent research advances in the fabrications of heterojunctions and composite photocatalysts. An inspiring review presented by Chen et al. [1] summarizes the application of transition metal dichalcogenides (TMDs)/graphene (GR) composites as co-catalysts in photocatalytic water splitting and pollutants degradation. It provides a comprehensive synopsis of the mechanism governing the enhanced photocatalytic activity of these TMDs/GR hybrids and will undoubtedly help in the development of the next generation of photocatalysts for solar energy utilization.

TiO_2_ and ZnO are well known as excellent UV-responsive semiconductors for efficient photocatalysis in the applications of contaminant detoxification and water splitting. A variety of strategies have been developed to extend the absorption range to the visible light region for better sunlight utilization. Ye et al. synthesized a series of g-C_3_N_4_/TiO_2_ photocatalysts for reductive degradation of polybromodiphenyl ethers [2]. g-C_3_N_4_/TiO_2_ with 2 wt % g-C_3_N_4_ loading showed the highest reaction rate, owing to the enhanced light absorption and effective charge carrier separation. In the work of Zhang et al. [3], p-Co_3_O_4_/n-TiO_2_ nanocomposite photocatalyst was prepared and used for visible-light-driven water splitting, showing a 25% higher H_2_ evolution rate than pure Co_3_O_4_ nanoparticles. Jiang et al. [4] reported on the first fabrication and characterization of TiO_2_ hollow spheres with spatially separated dual co-catalysts (Ag and RuO_2_). The resultant catalysts showed significantly enhanced activity towards H_2_ production under simulated sunlight. Yuan et al. [5] prepared g-C_3_N_4_ nanosheets/ZnO photocatalysts with different C_3_N_4_ loadings via a simple precipitation-calcination method. The enhanced performances of these catalysts in Cr(VI) reduction were ascribed to the increased visible light absorption and effective transfer and separation of photoproduced charge carriers. Li et al. [6] investigated the formation of ZnO nanowires doped with Mn^2+^ and Co^2+^ ions. This study reveals the importance of Mn^2+^ and Co^2+^ dopants in light absorption enhancement and inhibition of the recombination of electron-hole pairs.

Two articles in this special issue focus on the fabrication of composite photocatalysts consisting of ferrite or selenide. Xu et al. [7] demonstrated that Mn doping in the BiFeO_3_/BiFe_0.95_Mn_0.05_O_3_ and BiFe_0.95_Mn_0.05_O_3_ thin films can enable the formation of amounts of defects which result in poor photoactivity compared to pure BiFeO_3_ due to the higher carrier combination. Qiao et al. [8] prepared a novel Cu_1.8_Se/Cu_3_Se_2_ composite photocatalyst via a simple precipitation method, and it was found that the photocatalyst exhibited significantly enhanced photocatalysis under visible and even near-infrared light irradiations.

Bismuth oxyhalides, BiOX (X = Cl, Br, I), as a new generation of photocatalysts, have attracted considerable attention in designing novel composite photocatalysts. Liu et al. [9] synthesized three-dimensional flower-like BiOI/BiOX hybrids via a facile one-pot solvothermal approach. Among all tested BiOI/BiOX composites, BiOI/BiOCl appeared as the most efficient photocatalyst under both UV and visible light irradiations, due to its larger specific surface area and stronger light absorption capacity. The other work by Li et al. reports a novel heterostructure of multi-walled carbon nanotubes (MWCNTs) coated with BiOI nanosheets as an efficient photocatalyst [10]. This study provides valuable insights into the necessity of intimate interfacial contact for optimum charge carrier transfer and highlights the dominant roles of photo-induced holes (h^+^) and superoxide radicals (O_2_^•−^).

Finally, guest editors together with editorial assistant editors would like to thank all contributing authors for making this special issue a success.
